# The association between ethnicity, socioeconomic position and outcomes following initiation of methotrexate in juvenile idiopathic arthritis

**DOI:** 10.1016/j.ero.2025.10.001

**Published:** 2025-11-15

**Authors:** Richard P. Beesley, Lianne Kearsley-Fleet, Eileen Baildam, Michael W. Beresford, Sharon Douglas, Taunton R. Southwood, The UK JIA Biologics Register, Kimme L. Hyrich, Jenny H. Humphreys

**Affiliations:** 1 Versus Arthritis Centre for Epidemiology, Centre for Musculoskeletal Research, The University of Manchester, Manchester Academic Health Science Centre, Manchester, UK; 2 The Alexandra Hospital, Cheadle, Manchester, UK; 3 Department of Paediatric Rheumatology, Alder Hey Children’s NHS Foundation Trust, Liverpool, UK; 4 Institute of Life Course and Medical Sciences, University of Liverpool, Liverpool, UK; 5 Scottish Network for Arthritis in Children (SNAC), Isle of Arran, Scotland; 6 Institute of Child Health, University of Birmingham, Birmingham, UK; 7 National Institute of Health Research Manchester Biomedical Research Centre, Manchester University NHS Foundation Trust, Manchester, UK

## Abstract

**Objectives:**

This analysis investigates changes in disease activity and treatment persistence following initiation of methotrexate in children and young people with juvenile idiopathic arthritis (JIA), by ethnic group and socioeconomic position.

**Methods:**

Patients registered in the UK JIA Biologics Register starting methotrexate were included. Outcomes included a change in disease activity between treatment commencement and 6 months. Adjusted multivariable linear regression was used to assess the association between ethnicity or socioeconomic position. Treatment persistence was analysed using Kaplan-Meier estimates. Cox proportional hazards models were used to compare drug persistence between groups.

**Results:**

A total of 993 patients were included; 69% women, 88% White ethnic group (2% Mixed, 8% Asian, and 2% Black), and 24% in the most deprived socioeconomic group. Disease activity improved after 6 months for most children. There was no evidence that improvement differed by ethnicity or socioeconomic position.

The proportion of patients remaining on methotrexate at 12 months varied by ethnic group (61% of children with White ethnicity, 64% Asian, 65% Black, and 67% of children with Mixed ethnicity). The likelihood of stopping methotrexate over the 12-month period was similar between ethnicities and socioeconomic groups.

**Conclusions:**

Improvements in disease activity in the first 6 months following initiation of methotrexate in children and young people with JIA are similar between ethnic groups and independent of socioeconomic position. Although small sample sizes limit robust conclusions, further research to understand whether differences are seen following initiation of other therapies for JIA and the drivers of these differences is warranted.

## INTRODUCTION

Juvenile idiopathic arthritis (JIA) is a group of autoimmune disorders characterised by chronic joint inflammation of unknown aetiology with onset before 16 years old [1]. JIA is diagnosed in around 1 in 1600 children and young people. A 2023 publication also suggested there may be differences in the incidence and prevalence of JIA between different ethnic groups [2].

JIA may result in joint damage, chronic pain, and lifelong disability, with implications for long-term physical and mental health as well as social and economic prospects [3,4], although modern treatment approaches have resulted in better outcomes overall. There is felt to be a ‘window of opportunity’ in the treatment of JIA, in which early treatment and disease control are associated with better long-term outcomes [5]. Delays in diagnosis and/or treatment may have consequential impacts on disease activity and outcomes.

Following a diagnosis of JIA, treatment in the United Kingdom (UK) follows National Health Service (NHS) guidelines [6], with the majority of patients with polyarticular JIA, or whose disease is refractory to nonsteroidal antiinflammatory drugs and/or intraarticular steroid injections, commencing methotrexate as the current standard first-line disease-modifying treatment.

Not all children and young people with JIA experience disease control following initiation of methotrexate, with clinical response varying from 30% to 70% [7]. The reasons for this are multifactorial and may include factors associated with the drug-disease mechanism, concomitant drug therapy, disease severity at initiation, delays in initiation of treatment, experience of adverse events, or low adherence to treatment [8].

In other disease areas, cultural differences between communities have been identified as affecting healthcare-seeking behaviours [9], and in at least some circumstances, delays in accessing care have been associated with ethnicity [10]. Further, racism and discrimination impact on both health and healthcare systems [11]. Consequently, ethnicity may be associated with delays in diagnosis and initial treatment, associated with more severe disease at initial presentation and worse outcomes. Equally, socioeconomic position has been demonstrated to be associated with poor outcomes in JIA and other rheumatologic conditions [12,13]. The intersectionality between ethnicity and socioeconomic position may further compound these associations [14].

Socioeconomic position has also been shown to be associated with recorded response to disease-modifying antirheumatic drugs (DMARDs) in rheumatoid arthritis. In JIA, it is also conceivable that outcomes following treatment with methotrexate may be associated with a complex combination of biologic, environmental, and social factors. However, to date, outcomes following treatment with methotrexate in JIA have not been investigated by ethnic group and socioeconomic position.

This analysis aimed to investigate changes in disease activity and treatment persistence following initial treatment with methotrexate in children and young people with JIA in the UK, by ethnic group and socioeconomic position.

## METHODS

### Study design

This analysis used data collected as part of the UK JIA Biologics Register, which comprises the British Society for Paediatric and Adolescent Rheumatology Etanercept Cohort (BSPAR-ETN) and the Biologics for Children with Rheumatic Diseases (BCRD) studies, both identical in study design and data collection. Details of the UK JIA Biologics Register have been presented elsewhere [15]. In brief, since 2004, children with JIA have been recruited at the point of starting a biologic or JAK inhibitor therapy for their JIA or (between January 2004 and November 2017) initiating methotrexate, with the aim to investigate the safety and effectiveness of these therapies. This analysis is limited to children and young people starting methotrexate.

### Data collection

Baseline data were collected at the start of methotrexate treatment by the treating physician or affiliated clinical research nurse. Data items included patient demographics, JIA ILAR category [1], time since diagnosis, current disease activity captured using the JIA core outcome variables [16] and a pain visual analogue scale, and details of previous and current antirheumatic therapies.

Follow-up data were extracted from the patient’s medical record at 6 and 12 months after the start of the first registered treatment and then annually and included changes to antirheumatic drug therapy, including start/stop dates and reasons, the most recent recorded JIA core outcome variables, and adverse events. All data were captured as part of routine care, with no additional study visits for the purposes of data collection.

### Ethnicity

All patients in the NHS are asked to self-declare their ethnicity when they register with their general practitioner or hospital. Ethnic group in the UK JIA Biologics Register was captured at recruitment from that recorded in the child’s NHS hospital record, using a combination of both closed options and open-text descriptions. Responses were grouped according to the Office for National Statistics (ONS) England ethnic group classification [17]. This comprises 5 first-tier categories—White, Mixed, Asian, Black, and Other—although in this analysis, no patients had a recorded ethnic group of ‘Other’.

### Indices of deprivation

Home address postcodes were used to assign each patient to a nationwide deprivation rank using the most recent Indices of Multiple Deprivation (IMD) [18]. IMD is an indicator of relative multiple deprivations within small fixed geographic areas across the country (minimum population per area of 1000 people). IMD combines indicators from economic, social, and housing issues into a single deprivation score, which is ranked nationally. Seven separate weighted domain scores are combined to make the IMD, including income, employment, health deprivation, disability, education, skills, training, barriers to housing and services, crime, and living environment. Patient ranks were assigned to nationally determined quintiles of deprivation. As the calculation of IMD scores differs between nations in the UK, quintiles were determined separately for England (based on 2019 scores), Scotland (based on 2012 scores), and Wales (based on 2014 scores), then combined into an overall IMD quintile score; no patients were from Northern Ireland.

Due to the relatively small sample size overall, for analyses IMD was grouped into a dichotomous variable (the most deprived quintile compared to all other quintiles combined).

### Patient selection

The analyses included all patients starting methotrexate between January 2004 and November 2017, registered within 6 months of their methotrexate start date, for whom ethnicity was recorded, and at least 1 follow-up form had been completed. Data were extracted in January 2024.

The primary outcome was the change in 71-joint count Juvenile Arthritis Disease Activity Score (JADAS-71) [19] between baseline and 6 months. The JADAS-71 is a composite score of the Active Joint Count (AJC), Physician’s Global Assessment of overall disease activity (PGA), Patient/Parent Global Evaluation of overall wellbeing (PGE), and normalised erythrocyte sedimentation rate (ESR). Secondary outcomes included mean change in clinical (c)JADAS, each core outcome variable (AJC, limited joint count [LJC], PGA, PGE, Childhood Health Assessment Questionnaire of functional ability [CHAQ], ESR, and C-reactive protein), and patient/parent-reported levels of pain (scale 0-10), proportion of patients attaining minimal disease activity (MDA), and American College of Rheumatology (ACR) Paediatric Response (ACR-Pedi) of 30%, 50%, 70%, and 90%, and persistence on methotrexate over the first year of treatment. ACR-Pedi-30/50/70/90, indicators of improvement in overall disease activity, were modified to take into account low baseline scores for which small changes can have a disproportionate impact (Supplementary Table S1).

MDA [20] was considered attained as follows: persistent-oligoarthritis PGA ≤2.5cm and 0 AJC; all other ILAR subtypes PGA ≤3.4cm, PGE ≤2cm, and AJC ≤1. Enthesitis-related JIA was excluded, as MDA is not validated for these patients.

For analyses of disease activity, participants were also required to have at least 1 core outcome variable measured between 6 months before and up to 14 days after the start date of methotrexate. All participants contributed data to the analysis of treatment persistence.

Descriptive data, including demographics and disease characteristics (JADAS, cJADAS, and core outcome variables [COVs]) at baseline, are presented for the total study population, by ethnic group and by IMD group.

Multivariable linear regression was used to study the association between ethnicity, IMD, and change in JADAS-71 between baseline and 6 months. Only patients with complete ethnicity and IMD information were included in the model. The analysis was adjusted for potential confounders, selected *a priori* in advance, including gender, age, time between diagnosis and commencement of methotrexate, ILAR category, and baseline JADAS-71 score; an interaction term between ethnic group and IMD was also included in the model and the significance of the interaction term was assessed, which indicates whether the effect of IMD on change in JADAS-71 may differ depending on ethnic group.

Treatment persistence with methotrexate (without concomitant biologic DMARDS) was analysed using Kaplan-Meier estimates. Patients entered the model at the start of methotrexate and were considered to be on the drug until the earliest of date of stopping methotrexate (other than for remission), date of commencing concurrent treatment with another DMARD (eg, biologics), date of last follow-up, death, or 12 months from commencement of methotrexate. Patients were censored if they stopped methotrexate for remission. Kaplan-Meier curves of methotrexate persistence up to 12 months are presented, stratified by (1) ethnic group and (2) by IMD. The proportion of patients remaining on treatment at 12 months was estimated and compared between (1) ethnic groups and (2) IMD groups. Cox proportional hazards models were used to compare drug persistence between (1) ethnic groups and (2) IMD groups and adjusted for gender, age, time between diagnosis and commencement of methotrexate, ILAR category, and the interaction term between ethnicity and IMD.

Multiple imputation using chained equations (72 datasets) was used to account for missing covariates and disease activity data. Imputed variables included (at baseline and after 6 months) AJC, LJC, PGA, PGE, CHAQ, pain, and ESR. Complete variables included in the imputation model were gender, age, ILAR category, ethnicity, IMD, and time between diagnosis and commencement of methotrexate. JADAS-71 and cJADAS-71 at baseline and follow-up, JADAS-71 and cJADAS-71 change, and MDA and ACR-Pedi-30/50/70/90 were calculated using imputed values.

Analysis was performed using Stata Version 14.0.

### Patient involvement

Parents of children and young people with JIA, from a diverse range of ethnic backgrounds, took part in discussions with the authors prior to this analysis to help identify analysis priorities. This enabled the authors to develop the analysis plan, understand the needs of patients and families, and ensure the analyses reflected the interests and identities of a diverse population. Whether ethnicity is associated with disease outcomes was reported by families as a priority area for study.

## RESULTS

### Baseline characteristics

A total of 993 patients met the inclusion criteria, of which 69% were female, and the median age at start of methotrexate was 9 years (IQR: 4–13 years), with most starting within the first year following diagnosis (Table 1). Most of the patients were in the White ethnic group (88%), with 2% in the Mixed ethnic group, 8% in the Asian ethnic group, and 2% in the Black ethnic group. There were 24% patients in the most deprived IMD quintile. The most frequent ILAR category for all ethnic groups was RF-negative polyarthritis (33% of all patients, range 33%–40% by ethnic group). Mean initial dose of methotrexate was 13.1 mg/m^2^ (95% CI, 11.1-14.8); these were within expected clinical ranges. The proportion of patients of White ethnicity was slightly higher than national population estimates by ethnicity for children aged <16 years (Supplementary Table S2).

**Table 1:** Baseline characteristics of the 993 children and young people commencing methotrexate, for the drug persistence analysis

Characteristic		Whole cohort	Ethnic group				IMD group	
			White	Mixed	Asian	Black	Most deprived quintile	All others
N (row %)		993	870 (88)	24 (2)	79 (8)	20 (2)	208 (24)	663 (76)
Gender, n (%)	Male	305 (31)	270 (31)	6 (25)	22 (28)	7 (35)	66 (32)	203 (31)
	Female	688 (69)	600 (69)	18 (75)	57 (72)	13 (65)	142 (68)	460 (69)
Age at start of methotrexate	Median (IQR)	9 (4-13)	9 (4-13)	8 (4-13)	9 (4-14)	10 (6, 13)	9 (4-13)	9 (4-13)
Disease duration (time between diagnosis and commencement of methotrexate), y	Median (IQR)	0 (0-1)	0 (0-1)	0 (0-1)	0 (0-1)	0 (0, 2)	0 (0-1)	0 (0-1)
Indices of multiple deprivation (IMD) quintile, n (%)		N = 871						
	1—Most deprived	208 (24)	162 (21)	7 (37)	31 (45)	8 (44)	..	..
	2	161 (18)	136 (18)	<5	16 (23)	<5	..	..
	3	167 (19)	148 (19)	<5	12 (17)	<5	..	..
	4	173 (20)	165 (20)	<5	5 (7)	<5	..	..
	5—Least deprived	162 (10)	154 (20)	<5	5 (7)	<5	..	..
ILAR category, n (%)	Persistent oligo	192 (19)	166 (19)	6 (25)	15 (19)	5 (25)	36 (17)	143 (22)
	Oligo extended	161 (16)	150 (17)	<5	6 (8)	<5	30 (14)	109 (16)
	Systemic	46 (5)	41 (5)	<5	<5	<5	7 (3)	33 (5)
	Poly RF-	331 (33)	288 (33)	9 (38)	26 (33)	8 (40)	73 (35)	28 (33)
	Poly RF+	85 (9)	63 (7)	<5	14 (18)	<5	23 (11)	49 (7)
	Psoriatic	78 (8)	72 (8)	<5	6 (8)	<5	17 (8)	49 (7)
	Enthesitis-related	71 (7)	64 (7)	<5	6 (8)	<5	14 (7)	47 (7)
	Undifferentiated	29 (3)	26 (3)	<5	<5	<5	8 (4)	15 (2)
History of chronic anterior uveitis at start of treatment, n (%)	Yes	92 (10)	80 (10)	0 (0)	7 (10)	5 (26)	14 (7)	67 (11)
	No	839 (90)	736 (90)	23 (100)	66 (90)	14 (74)	176 (93)	562 (89)

IMD, index of multiple deprivation; <5 indicates fewer than 5 cases.

Of these, 810 patients met the inclusion criteria for analyses of disease activity, of which 70% were female, 87% were in the White ethnic group (3% Mixed, 8% Asian, and 2% Black), and 24% were in the most deprived IMD quintile (Supplementary Table S3). Missingness for these 810 patients is presented in Supplementary Table S4.

### Outcomes following initial methotrexate treatment

After 6 months of methotrexate treatment, disease activity had improved for all ethnic groups (Table 2), with an overall mean change in JADAS at 6 months (95% CI) of −10.5 units (−11.4 to −9.5) for patients of White ethnicity, −16.6 (−23.4 to −9.9) for patients of Mixed ethnicity, −11.4 (−14.1 to −8.6) for patients of Asian ethnicity, and −7.9 (−16.6 to 0.9) for patients of Black ethnicity. However, for Black patients, the 95% CIs included the null hypothesis of 0. Patients from both the most deprived IMD group and all other IMD groups showed similar improvement in JADAS (most deprived quintile −10.0 [−12.0 to −8.1]; all others −10.3 [−11.3 to −9.3]).

**Table 2:** Change in JADAS between baseline and 6 months after initiation of methotrexate amongst the 810 children and young people with JIA by ethnic group and deprivation

Variable	Ethnic Group				IMD group	
	White	Mixed	Asian	Black	Most deprived quintile	All others
N	702	22	68	18	168	543
JADAS at baseline, mean (95% CI)	16.8 (15.9-17.6)	21.9 (15.3-28.6)	17.7 (14.9-20.5)	18.9 (13.7-24.0)	17.5 (15.7-19.2)	16.4 (15.4-17.3)
JADAS at 6 months, mean (95% CI)	6.3 (5.7-6.8)	5.3 (2.5-8.1)	6.3 (4.6-8.1)	11.0 (5.1-17.0)	7.4 (6.2-8.6)	6.1 (5.4-6.7)
Change in JADAS, mean (95% CI)	−10.5 (−11.4 to −9.5)	−16.6 (−23.4 to −9.9)	−11.4 (−14.1 to −8.6)	−7.9 (−16.6 to 0.9)	−10.0 (−12.0 to −8.1)	−10.3 (−11.3 to −9.3)
Relative mean changes in JADAS (95% CI)	Reference	−6.2 (−11.6 to −0.7)	−0.9 (−4.1 to 2.3)	2.6 (−3.7 to 9.0)	0.3 (−1.9 to 2.4)	Reference
Adjusted relative mean change in JADAS (95% CI)	Reference	−1.2 (−6.1 to 3.7)	−0.4 (−3.1 to 2.3)	3.3 (−2.7 to 9.3)	1.3 (−0.2 to 2.7)	Reference
**At 6 months:**						
MDA	45 (41-49)	57 (35-79)	43 (29-56)	38 (13-63)	40 (32-48)	46 (41-51)
ACR-Pedi-30 response	37 (34-41)	45 (23-66)	49 (37-61)	22 (3-41)	40 (33-48)	37 (33-42)
ACR-Pedi-50 response	36 (32-39)	42 (31-64)	45 (33-58)	22 (3-41)	37 (30-45)	36 (32-40)
ACR-Pedi-70 response	31 (28-35)	41 (20-63)	36 (25-48)	11 (0-26)	29 (22-36)	32 (28-36)
ACR-Pedi-90 response	25 (22-29)	35 (15-56)	27 (16-38)	11 (0-26)	21 (14-27)	26 (23-30)

IMD, index of multiple deprivation; JADAS, Juvenile Arthritis Disease Activity Score; MDA, minimal disease activity.

Adjusted relative mean change adjusted for age, gender, grouped ILAR subclass, IMD group, and time between diagnosis and commencement of methotrexate monotherapy.

Similarly, all ethnic groups showed an improvement in individual COVs (Supplementary Table S5), although the IQR for all COVs for Black patients included the null hypothesis of 0, with the exception of ESR. Patients from both the most deprived IMD group and all other IMD groups showed improvements in all individual COVs. The proportion of children and young people (Table 2) who had MDA after 6 months of treatment was 45% (95% CI, 41%-49%), ranging from 38% to 57% by ethnic group. The proportion of children and young people achieving ACR-Pedi-30 was 38% (95% CI, 35%-42%), varying from 22% (95% CI, 3%-41%) for children with Black ethnicity to 49% (95% CI, 37%-61%) for children with Asian ethnicity. The proportion achieving ACR-Pedi-90 varied from 11% (95% CI, 0%-26%) for children with Black ethnicity to 35% (95%CI, 15%-56%) for children with Mixed ethnicity.

In the multivariable linear regression (Supplementary Table S6; 711 patients with complete IMD data available included in the model), investigating change in JADAS after 6 months of methotrexate therapy, neither ethnicity nor IMD, were statistically significantly associated with change in JADAS score. The interaction between ethnicity and IMD was not significant. Older age was associated with less improvement in JADAS, and higher baseline JADAS was associated with a greater reduction in JADAS at 6 months.

### Persistence of methotrexate

**Figure 1:**
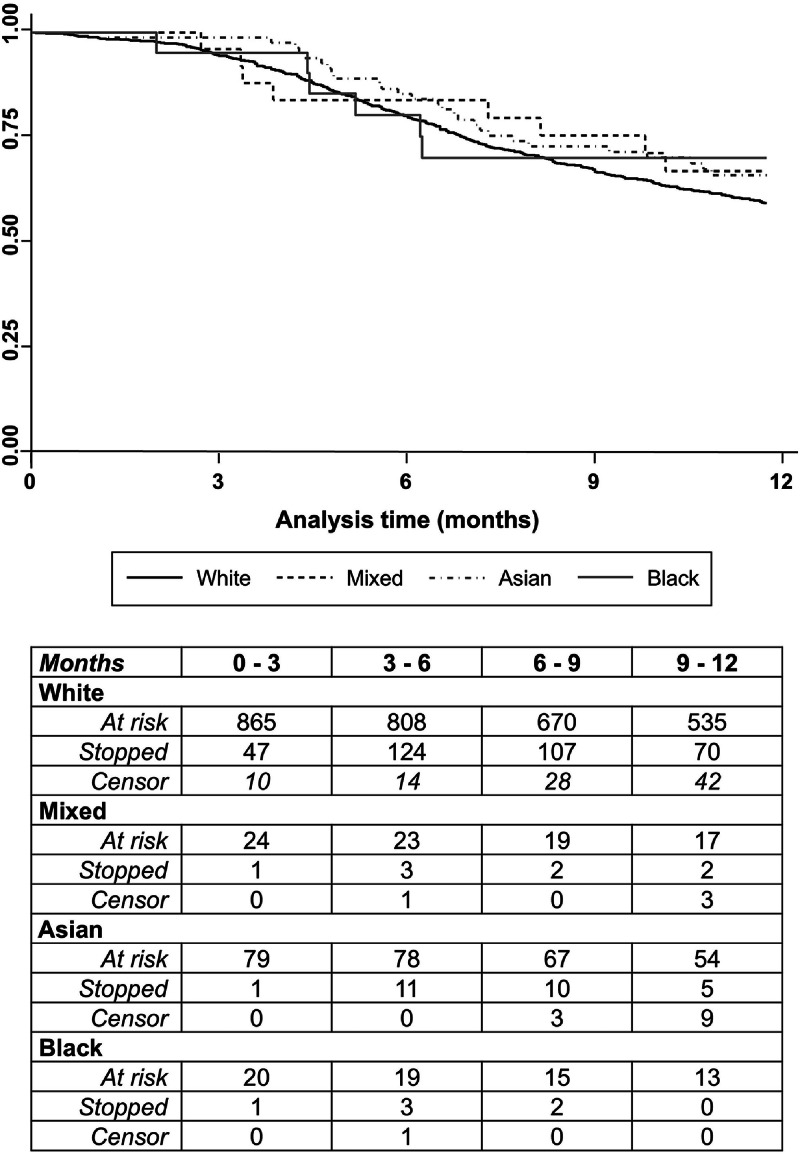
Kaplan-Meier survival curve for methotrexate monotherapy by ethnic group for children and young people with JIA.

**Figure 2:**
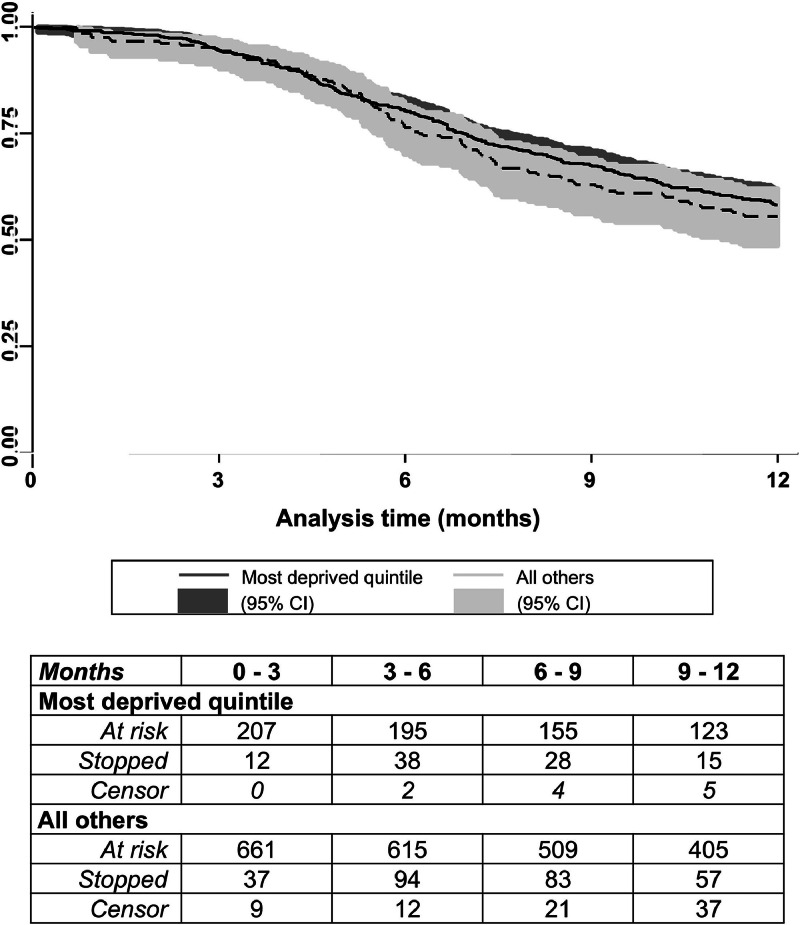
Kaplan-Meier survival curve for methotrexate monotherapy by Index of Deprivation group for children and young people with JIA.

All 993 patients were included in the persistence of methotrexate as monotherapy analysis. Figure 1 shows the Kaplan-Meier survival curve for methotrexate persistence up to 12 months, comparing children by ethnic group; Figure 2 shows this by IMD group. Overall, 61% of patients remained on methotrexate monotherapy at 1 year (95% CI, 60%-63%), with a median treatment survival of 1.0 year (95% CI, 0.5-1.5 years). The proportion of patients (Table 3) remaining on methotrexate monotherapy at 12 months ranged from 61% of children with White ethnicity (95% CI, 59%-62%) and 64% of children with Asian ethnicity (95% CI, 60%-68%) to 65% of children with Black ethnicity (95% CI, 56%-74%) and 67% of children with Mixed ethnicity (95% CI, 60%-74%). The likelihood of stopping methotrexate monotherapy over the 12-month period was similar between ethnicities and IMD groups.

**Table 3:** Reasons for stopping methotrexate as monotherapy within 12 months and hazard ratio for survival of methotrexate monotherapy by ethnic group and IMD

	Ethnic group				IMD Group	
	White	Mixed	Asian	Black	Most deprived quintile	All others
Total	870	24	79	20	208	663
Percentage remaining on methotrexate as monotherapy (95% CI)	61 (59-62)	67 (60-74)	64 (60-68)	65 (56-74)	58 (55-61)	61 (60-63)
Of those discontinuing MTX as monotherapy:						
Percentage stopping MTX due to remission	5	0	7	0	2	6
Percentage stopping MTX not for remission	41	50	26	33	42	38
Percentage continuing MTX and adding biologic therapy	54	50	67	67	56	56
Unadjusted hazard ratio (95% CI)	Reference	0.79 (0.39-1.60)	0.79 (0.53-1.17)	0.73 (0.33-1.65)	1.10 (0.87-1.39)	Reference
Adjusted hazard ratio (95% CI)	Reference	0.97 (0.46-2.07)	0.73 (0.49-1.10)	0.61 (0.25-1.47)	1.15 (0.90-1.46)	Reference

Adjusted hazard ratio adjusted for age, gender, grouped ILAR subclass, IMD group, and time between diagnosis and commencement of methotrexate monotherapy.

## DISCUSSION

This study investigated changes, for the first time, in disease activity and treatment persistence following methotrexate therapy in children and young people with JIA in the UK by ethnic group and socioeconomic position. Overall, children and young people with JIA showed improvements in JADAS and core outcome variables after 6 months of treatment. Reassuringly, most patients improved in their disease activity, with no evidence that it differed significantly by ethnicity or socioeconomic position in this analysis. In addition, >60% of patients remained on methotrexate monotherapy at 1 year. Neither ethnicity nor socioeconomic position was associated with staying on treatment.

Similar to our findings, Soulsby et al [21] reported that deprivation was not associated with disease activity. However, their study found that high levels of community poverty were associated with persistent functional disability. Additionally, they observed that 'non-White' ethnicity was associated with high disease activity, suggesting that the relationship between ethnicity and disease activity may be independent of poverty and deprivation.

In contrast to their study, our work focused on change in disease activity over time rather than cross-sectional levels of disease activity and found no association that improvement in disease activity was associated with deprivation or ethnicity and included all subtypes of JIA rather than solely polyarticular JIA.

Shoop-Worrall et al [7] investigated change in disease activity over time in a longitudinal multivariable analysis of children and young people with JIA in the UK. Ethnicity was not the primary focus of the study but was found to be associated with membership of the subgroup of patients in whom patient/parent measures of wellbeing (ie, PGE) remained persistently high despite overall improved measures of disease activity [7]. In our analysis, we did not formally assess whether there was a statistical difference in wellbeing alone, although it forms part of JADAS.

In contrast to our study, several international studies have reported associations between ethnicity and functional outcomes. In Canada, Oen et al [22] found that Canadian Indigenous children with juvenile arthritis aged >8 with >5 years follow-up were found to have worse functional ability (higher CHAQ score) than Canadian non-Indigenous children. In the USA [23], Hispanic children have been found to have higher CHAQ scores than non-Hispanic children, although both groups had low scores overall, indicating relatively good functional ability in all children. Similarly, ethnicity has been associated with an increased risk of joint damage, pain, and functional disability [24]. Those studies did not measure change in disease activity over time, only at a single cross-sectional time point whilst on treatment, although the follow-up data in our study showed all ethnic groups experienced improving measures of disease activity (including both CHAQ and pain) over time; with the exception of children with Black ethnicity, for whom there was a trend towards improvement but it did not reach statistical significance.

Lower socioeconomic position has been demonstrated to be associated with poor outcomes in JIA [12] and impaired quality of life [25], although the potential interaction between socioeconomic position and ethnicity was not previously considered. However, our analysis did not find any association with socioeconomic position and improvement in disease activity, and quality of life was not investigated.

The strengths of this study include that it is based on data from a national cohort study of children and young people with JIA starting methotrexate. All participants are systematically followed up over time, with their disease activity measured at regular time points. Whilst inclusion into the cohort study is optional, patients who agreed to take part are considered broadly representative of the whole JIA population. Our study is limited by the relatively small sample size for some ethnic groups, and the interaction between IMD and ethnicity results in some very small group sizes. This leads to increased variability and decreased statistical power, so there is insufficient data to make broader conclusions. There was also a high level of missing data in some outcome measures, which may have affected the precision of the estimates; the impact may be more substantial in smaller ethnic subgroups. The use of multiple imputation sought to reduce the selection bias introduced by this limitation. In addition, there is no record of symptom onset (only of date of diagnosis), so the potential impact of any delays in initial diagnosis cannot be identified. However, within the data available in this national register, there does not appear to be any significant delays in the commencement of methotrexate after diagnosis. Ethnicity data are self-reported to clinical administrators within the health service, and our study extracted that data from the NHS record; this assumes ethnicity is recorded accurately in those records and that patients report their ethnicity in a consistent and repeatable way. However, there are multiple reasons why this may not be done.

Overall, it appears that in this group of children and young people with JIA, most patients improved in their disease activity in the first 6 months following methotrexate treatment. It is reassuring that there is no evidence that this differed by ethnicity or socioeconomic position, and there is no evidence that the effects of socioeconomic position are moderated by ethnicity or vice versa. Nevertheless, the interaction between ethnicity, socioeconomic position, and disease outcomes is likely to be complex and not fully explainable through this analysis. Further research to understand whether differences are seen following initiation of other therapies for JIA and the drivers of these differences is warranted.

## Competing interests

RB is the founder and unpaid Director of the charity Juvenile Arthritis Research. None of the authors have any conflicts of interest with respect to this paper.

## Appendix Supplementary materials

Image, application 1

## Footnotes

Supplementary material associated with this article can be found, in the online version, at doi:10.1016/j.ero.2025.10.001.
