# Supplementary material for: The association between ethnicity, socioeconomic position and outcomes following initiation of methotrexate in juvenile idiopathic arthritis
Source: EULAR Rheumatol Open. 2025 Nov 15;1(4):372–9. doi: 10.1016/j.ero.2025.10.001 (PMC13292529; doi:10.1016/j.ero.2025.10.001)
Supplement: Supplementary file 1 [file mmc1.docx]

# Supplementary tables

Supplementary Table S1. Modification of ACR-Pedi-30/50/70/90 to take account of low baseline scores.

| **Component** | **Original ACR-Pedi-30** | **Modified ACR-Pedi-30 for analyses** |
| --- | --- | --- |
| PGA | Change in value between baseline and follow-up assessment. | As original, except a score of 0 at both baseline and follow-up was considered to be an improvement of at least 30%; a score of 0 at baseline and ≥0 at follow-up is considered to be a worsening of more than 30%. |
| PGE | Change in value between baseline and follow-up assessment. | As original, except a score of 0 at both baseline and follow-up was considered to be an improvement of at least 30%; a score of 0 at baseline and ≥0 at follow-up is considered to be a worsening of more than 30%. |
| AJC | Change in value between baseline and follow-up assessment. | As original, except a score of 0 at both baseline and follow-up was considered to be an improvement of at least 30%; a score of 0 at baseline and ≥0 at follow-up is considered to be a worsening of more than 30%. |
| LJC | Change in value between baseline and follow-up assessment. | As original, except a score of 0 at both baseline and follow-up was considered to be an improvement of at least 30%; a score of 0 at baseline and ≥0 at follow-up is considered to be a worsening of more than 30%. |
| CHAQ | Change in value between baseline and follow-up assessment. | As original, except a score of 0 at both baseline and follow-up was considered to be an improvement of at least 30%; a score of 0 at baseline and ≥0 at follow-up is considered to be a worsening of more than 30%. |
| ESR | Change in value between baseline and follow-up assessment. | Values <20mm/hr were considered normal. Any change within this normal range, regardless of value, was considered as an improvement.  If follow-up is ≥20mm/hour when baseline had been <20mm, then % worsening is calculated from 20mm/hour. Values ≥20mm at baseline are treated as face value and response/worsening calculated appropriately. |
| ACR-Pedi-30 | Proportion of patients improvement between baseline and follow-up assessment in at least three of PGA, PGE, AJC, LJC, CHAQ, ESR; with no more than 1 remaining variable worsening by more than 30%. | Proportion of patients improvement between baseline and follow-up assessment in at least three of PGA, PGE, AJC, LJC, CHAQ, ESR; with no more than 1 remaining variable worsening by more than 30%, using modified variables above. |

PGA - Physician Global Assessment of disease activity. PGE - Patient/parent Global Evaluation of wellbeing. AJC - Active Joint Count. LJC - Limited Joint Count. CHAQ - Childhood Health Assessment Questionnaire (CHAQ). ESR - Erythrocyte Sedimentation Rate. ACR-Pedi-50/70/90 differ from ACR-Pedi-30 in that at least three variables must show improvement of at least 50%, 70% or 90% respectively compared to baseline, with no more than 1 remaining variable worsening by more than 30%.

Supplementary Table S2. Proportion, by ethnic group, of children and young commencing methotrexate therapy compared to national population estimates and the estimated proportion of children and young people with JIA.

|  | **White** | **Mixed** | **Asian** | **Black** |
| --- | --- | --- | --- | --- |
| England & Wales, under 16s* | 73% | 7% | 12% | 5% |
| Incident JIA cases** | 83% | 4% | 6% | 3% |
| This analysis | 88% | 2% | 8% | 2% |

* England & Wales national population estimates for under 16s from 2011 Census (ONS). ** Proportion of JIA cases by ethnic group^2^.

Supplementary Table S3. Baseline characteristics of the 810 children and young people commencing methotrexate and included in disease activity analysis.

| **Characteristic** | | | **Whole cohort** | | **Ethnic group** | | | | | | | |  | | **IMD group** | | | |
| --- | --- | --- | --- | --- | --- | --- | --- | --- | --- | --- | --- | --- | --- | --- | --- | --- | --- | --- |
|  |  |  |  |  | **White** | | **Mixed** | | **Asian** | | **Black** | |  | | **Most deprived quintile** | | **All others** | |
| n (row %) |  | 810 | | 702 (87) | | 22 (3) | | 68 (8) | | 18 (2) | |  | | 168 (24) | | 543 (76) | |  |
| Gender, n (%) | Male | 245 (30) | | 214 (30) | | 6 (27) | | 19 (28) | | 6 (33) | |  | | 54 (32) | | 167 (31) | |  |
|  | Female | 565 (70) | | 488 (70) | | 16 (73) | | 49 (72) | | 12 (67) | |  | | 114 (68) | | 376 (69) | |  |
| Age at start of methotrexate | Median (IQR) | 9 (4, 13) | | 9 (4, 12) | | 8 (4, 13) | | 9 (4, 14) | | 10 (5, 13) | |  | | 9 (4, 13) | | 9 (4, 12) | |  |
| Disease duration (time between diagnosis and commencement of methotrexate), years | Median (IQR) | 0 (0, 1) | | 0 (0, 1) | | 0 (0, 1) | | 0 (0, 1) | | 0 (0, 2) | |  | | 0 (0, 1) | | 0 (0, 1) | |  |
| Indices of Multiple Deprivation (IMD) quintile, n (%) | 1 – Most deprived | 168 (24) | | 127 (21) | | 7 (41) | | 27 (45) | | 7 (44) | |  | | .. | | .. | |  |
|  | 2 | 129 (18) | | 105 (17) | | < 5 | | 15 (25) | | < 5 | |  | | .. | | .. | |  |
|  | 3 | 146 (21) | | 131 (21) | | < 5 | | 9 (15) | | < 5 | |  | | .. | | .. | |  |
|  | 4 | 138 (19) | | 132 (21) | | < 5 | | < 5 | | < 5 | |  | | .. | | .. | |  |
|  | 5 – Least deprived | 130 (18) | | 123 (20) | | < 5 | | < 5 | | < 5 | |  | | .. | | .. | |  |
| ILAR category, n (%) | Persistent oligo | 158 (20) | | 136 (19) | | < 5 | | 13 (19) | | < 5 | |  | | 29 (17) | | 116 (21) | |  |
|  | Oligo extended | 132 (16) | | 124 (18) | | < 5 | | 5 (7) | | < 5 | |  | | 22 (13) | | 91 (17) | |  |
|  | Systemic | 32 (5) | | 27 (4) | | < 5 | | < 5 | | < 5 | |  | | < 5 | | 24 (4) | |  |
|  | Poly RF- | 277 (34) | | 238 (34) | | 9 (41) | | 22 (32) | | 8 (44) | |  | | 63 (38) | | 184 (34) | |  |
|  | Poly RF+ | 61 (8) | | 43 (6) | | < 5 | | 10 (15) | | < 5 | |  | | 17 (10) | | 35 (6) | |  |
|  | Psoriatic | 65 (8) | | 59 (8) | | < 5 | | 6 (9) | | < 5 | |  | | 15 (9) | | 41 (8) | |  |
|  | Enthesitis-related | 59 (7) | | 52 (7) | | < 5 | | 6 (9) | | < 5 | |  | | 11 (7) | | 39 (7) | |  |
|  | Undifferentiated | 26 (3) | | 23 (3) | | < 5 | | < 5 | | < 5 | |  | | < 5 | | 13 (2) | |  |
| History of chronic anterior uveitis at start of treatment, n (%) | Yes | 77 (10) | | 68 (10) | | < 5 | | 6 (10) | | < 5 | |  | | 9 (6) | | 58 (11) | |  |
|  | No | 692 (90) | | 601 (90) | | 21 (100) | | 56 (90) | | 14 (82) | |  | | 149 (94) | | 89 (464) | |  |

IQR – Interquartile range. Time to registration – time (years) between diagnosis of JIA and registration and commencement of treatment. IMD – Index of Multiple Deprivation. <5 indicates fewer than 5 cases.

Supplementary Table S4. Proportion of missing data from demographic and core outcome variables of the 810 children and young people commencing methotrexate and included in the disease activity analysis.

| **Variable** | **Ethnic group** | | | |  | **IMD group** | | |
| --- | --- | --- | --- | --- | --- | --- | --- | --- |
|  | **White** | **Mixed** | **Asian** | **Black** |  | **Most deprived quintile** | **All others** |  |
| Number of patients | 702 | 22 | 68 | 18 |  | 168 | 543 |  |
| Gender | 0% | 0% | 0% | 0% |  | 0% | 0% |  |
| Age at start of methotrexate | 0% | 0% | 0% | 0% |  | 0% | 0% |  |
| Index of multiple deprivation (IMD) | 12% | 23% | 12% | 11% |  | 0% | 0% |  |
| ILAR category | 0% | 0% | 0% | 0% |  | 0% | 0% |  |
| History of chronic anterior uveitis at start of treatment | 5% | 5% | 9% | 6% |  | 6% | 4% |  |
| Disease duration | 1% | 0% | 0% | 0% |  | 2% | 1% |  |
|  |  |  |  |  |  |  |  |  |
| JADAS (calculated) |  |  |  |  |  |  |  |  |
| Baseline | 54% | 64% | 57% | 50% |  | 55% | 55% |  |
| 6 Months | 54% | 59% | 46% | 44% |  | 57% | 51% |  |
|  |  |  |  |  |  |  |  |  |
| cJADAS (calculated) |  |  |  |  |  |  |  |  |
| Baseline | 44% | 55% | 46% | 44% |  | 40% | 47% |  |
| 6 Months | 40% | 41% | 34% | 39% |  | 39% | 40% |  |
|  |  |  |  |  |  |  |  |  |
| AJC |  |  |  |  |  |  |  |  |
| Baseline | 3% | 5% | 0% | 0% |  | 4% | 3% |  |
| 6 Months | 11% | 9% | 13% | 22% |  | 11% | 12% |  |
|  |  |  |  |  |  |  |  |  |
| LJC |  |  |  |  |  |  |  |  |
| Baseline | 5% | 5% | 6% | 0% |  | 7% | 6% |  |
| 6 Months | 11% | 9% | 13% | 22% |  | 11% | 12% |  |
|  |  |  |  |  |  |  |  |  |
| PGA |  |  |  |  |  |  |  |  |
| Baseline | 33% | 32% | 41% | 44% |  | 32% | 36% |  |
| 6 Months | 28% | 32% | 24% | 28% |  | 28% | 28% |  |
|  |  |  |  |  |  |  |  |  |
| PGE |  |  |  |  |  |  |  |  |
| Baseline | 30% | 41% | 28% | 28% |  | 29% | 32% |  |
| 6 Months | 32% | 23% | 28% | 39% |  | 30% | 31% |  |
|  |  |  |  |  |  |  |  |  |
| Pain |  |  |  |  |  |  |  |  |
| Baseline | 32% | 41% | 25% | 33% |  | 31% | 33% |  |
| 6 Months | 33% | 23% | 29% | 33% |  | 30% | 33% |  |
|  |  |  |  |  |  |  |  |  |
| CHAQ |  |  |  |  |  |  |  |  |
| Baseline | 32% | 32% | 25% | 22% |  | 30% | 32% |  |
| 6 Months | 31% | 27% | 26% | 33% |  | 26% | 31% |  |
|  |  |  |  |  |  |  |  |  |
| ESR |  |  |  |  |  |  |  |  |
| Baseline | 17% | 9% | 15% | 6% |  | 20% | 14% |  |
| 6 Months | 25% | 27% | 25% | 22% |  | 24% | 23% |  |

cJADAS - clinical Juvenile Arthritis Disease Activity Score (cJADAS-71). AJC - Active Joint Count. LJC - Limited Joint Count. PGA - Physician Global Assessment of disease activity. PGE - Patient/parent Global Evaluation of wellbeing. CHAQ - Childhood Health Assessment Questionnaire (CHAQ). ESR - Erythrocyte Sedimentation Rate. IMD – Index of Multiple Deprivation. MDA – Minimal Disease Activity.Supplementary Table S5. Measures of disease activity at baseline and six months after commencement of methotrexate of the 810 children and young people with JIA, by ethnic group and deprivation.

| **Disease activity measure, mean (95% CI)** | **Ethnic group** | | | | |  | | **IMD Group** | |
| --- | --- | --- | --- | --- | --- | --- | --- | --- | --- |
|  | **White** | **Mixed** | **Asian** | **Black** |  | | **Most deprived quintile** | | **All others** |
| **N** | 702 | 22 | 68 | 18 |  | | 168 | | 543 |
|  |  |  |  |  |  | |  | |  |
| **cJADAS** |  |  |  |  |  | |  | |  |
| Baseline | 15.6 (14.8, 16.5) | 20.4 (13.8, 26.9) | 15.4 (12.7, 18.1) | 16.7 (12.3, 21.1) |  | | 16.2 (14.5, 17.8) | | 15.2 (14.3, 16.1) |
| 6 Months | 6.0 (5.4, 6.5) | 4.9 (2.3, 7.4) | 6.0 (4.3, 7.6) | 10.4 (4.7, 16.2) |  | | 7.1 (5.9, 8.3) | | 5.8 (5.2, 6.4) |
| Change | -9.7 (-10.6, -8.8) | -15.5 (-22.2, -8.8) | -9.5 (-12, -6.9) | -6.3 (-14.1, 1.5) |  | | -9 (-10.9, -7.2) | | -9.5 (-10.5, -8.5) |
|  |  |  |  |  |  | |  | |  |
| **AJC** |  |  |  |  |  | |  | |  |
| Baseline | 7.6 (6.9, 8.2) | 10.7 (5.7, 15.7) | 7.3 (5.3, 9.2) | 8.1 (4.8, 11.3) |  | | 8 (6.6, 9.3) | | 7.1 (6.4, 7.8) |
| 6 Months | 1.9 (1.6, 2.2) | 1.5 (0.2, 2.7) | 1.8 (0.9, 2.6) | 4.5 (0.6, 8.4) |  | | 2.4 (1.7, 3.1) | | 1.8 (1.5, 2.2) |
| Change | -5.6 (-6.3, -5) | -9.2 (-14.4, -4.1) | -5.5 (-7.3, -3.7) | -3.6 (-8.8, 1.7) |  | | -5.5 (-6.9, -4.2) | | -5.3 (-6, -4.6) |
|  |  |  |  |  |  | |  | |  |
| **LJC** |  |  |  |  |  | |  | |  |
| Baseline | 6.1 (5.4, 6.7) | 7.3 (2.6, 12.1) | 5.5 (3.6, 7.4) | 7.3 (4, 10.6) |  | | 5.9 (4.8, 7) | | 5.7 (5, 6.4) |
| 6 Months | 2.1 (1.7, 2.4) | 0.9 (0.1, 1.7) | 1.8 (0.9, 2.8) | 4.4 (0.8, 8) |  | | 2.2 (1.5, 2.9) | | 2 (1.6, 2.3) |
| Change | -4.0 (-4.6, -3.4) | -6.5 (-11.3, -1.6) | -3.7 (-5.3, -2.1) | -2.9 (-8.1, 2.4) |  | | -3.7 (-4.8, -2.7) | | -3.7 (-4.4, -3.1) |
|  |  |  |  |  |  | |  | |  |
| **PGA** |  |  |  |  |  | |  | |  |
| Baseline | 3.9 (3.7, 4.1) | 4.4 (3.2, 5.7) | 3.9 (3.2, 4.5) | 3.7 (2.5, 5) |  | | 3.9 (3.5, 4.2) | | 3.9 (3.7, 4.1) |
| 6 Months | 1.5 (1.4, 1.7) | 1.2 (0.4, 2) | 1.6 (1.1, 2.1) | 2.6 (1.3, 3.9) |  | | 1.8 (1.4, 2.1) | | 1.5 (1.3, 1.7) |
| Change | -2.4 (-2.6, -2.1) | -3.2 (-4.7, -1.8) | -2.3 (-2.9, -1.6) | -1.1 (-3, 0.7) |  | | -2.1 (-2.6, -1.6) | | -2.4 (-2.7, -2.1) |
|  |  |  |  |  |  | |  | |  |
| **PGE** |  |  |  |  |  | |  | |  |
| Baseline | 4.2 (3.9, 4.4) | 5.2 (3.8, 6.6) | 4.3 (3.5, 5) | 4.9 (3.5, 6.3) |  | | 4.3 (3.9, 4.8) | | 4.2 (3.9, 4.5) |
| 6 Months | 2.5 (2.3, 2.8) | 2.2 (1.1, 3.3) | 2.6 (1.9, 3.2) | 3.4 (1.9, 4.8) |  | | 2.9 (2.5, 3.4) | | 2.4 (2.2, 2.7) |
| Change | -1.7 (-2, -1.4) | -3.0 (-4.5, -1.5) | -1.7 (-2.6, -0.8) | -1.6 (-3.6, 0.4) |  | | -1.4 (-2, -0.8) | | -1.8 (-2.1, -1.4) |
|  |  |  |  |  |  | |  | |  |
| **Pain** |  |  |  |  |  | |  | |  |
| Baseline | 4.5 (4.2, 4.7) | 5.5 (4.2, 6.8) | 4.5 (3.7, 5.3) | 4.3 (2.8, 5.8) |  | | 4.6 (4.1, 5.2) | | 4.4 (4.1, 4.7) |
| 6 Months | 2.6 (2.4, 2.8) | 2.5 (1.3, 3.7) | 2.2 (1.6, 2.8) | 3.5 (1.9, 5) |  | | 3 (2.5, 3.4) | | 2.5 (2.2, 2.7) |
| Change | -1.9 (-2.2, -1.5) | -3.0 (-4.6, -1.5) | -2.3 (-3.1, -1.4) | -0.9 (-3, 1.3) |  | | -1.7 (-2.3, -1) | | -1.9 (-2.3, -1.6) |
|  |  |  |  |  |  | |  | |  |
| **CHAQ** |  |  |  |  |  | |  | |  |
| Baseline | 1.0 (0.9, 1.1) | 1.1 (0.7, 1.5) | 1.1 (0.9, 1.3) | 1.1 (0.6, 1.6) |  | | 1.1 (1, 1.2) | | 1 (0.9, 1) |
| 6 Months | 0.7 (0.6, 0.7) | 0.7 (0.3, 1) | 0.6 (0.4, 0.8) | 0.8 (0.3, 1.2) |  | | 0.7 (0.6, 0.9) | | 0.6 (0.5, 0.7) |
| Change | -0.3 (-0.4, -0.3) | -0.4 (-0.8, 0) | -0.5 (-0.7, -0.3) | -0.3 (-0.9, 0.2) |  | | -0.4 (-0.5, -0.2) | | -0.4 (-0.4, -0.3) |
|  |  |  |  |  |  | |  | |  |
| **ESR** |  |  |  |  |  | |  | |  |
| Baseline | 24.3 (22.3, 26.3) | 31.1 (18.8, 43.4) | 39.2 (30.7, 47.6) | 38.2 (23.7, 52.7) |  | | 27.4 (22.9, 31.8) | | 24.9 (22.6, 27.1) |
| 6 Months | 12.2 (11, 13.5) | 14.8 (6.8, 22.8) | 14.3 (9.2, 19.5) | 20.2 (11.4, 29.1) |  | | 13.4 (10.7, 16.2) | | 12.2 (10.8, 13.6) |
| Change | -12.1 (-14.1, -10.1) | -16.3 (-26.8, -5.9) | -24.8 (-32.7, -16.9) | -18.0 (-33.5, -2.4) |  | | -13.9 (-18.5, -9.4) | | -12.7 (-14.9, -10.5) |

cJADAS - clinical Juvenile Arthritis Disease Activity Score (cJADAS-71). AJC - Active Joint Count. LJC - Limited Joint Count. PGA - Physician Global Assessment of disease activity. PGE - Patient/parent Global Evaluation of wellbeing. CHAQ - Childhood Health Assessment Questionnaire (CHAQ). ESR - Erythrocyte Sedimentation Rate. IMD – Index of Multiple Deprivation.

Supplementary Table S6. Relative mean change in JADAS between baseline and 6-month follow-up in the 810 children and young people with JIA treated with methotrexate, by ethnic group and deprivation.

| **Variable** | **Coefficient (95% CI)** |
| --- | --- |
| Ethnic group |  |
| White | Reference |
| Mixed | -1.2 (-6.1, 3.7) |
| Asian | -0.4 (-3.1, 2.3) |
| Black | 3.3 (-2.7, 9.3) |
| IMD |  |
| Most deprived quintile | 1.3 (-0.2, 2.7) |
| All other quintiles | Reference |
| Ethnicity / IMD interaction |  |
| Mixed / All other quintiles | -2.5 (-9.7, 4.8) |
| Asian / all other quintiles | -0.7 (-4.9, 3.6) |
| Black / all other quintiles | 2.8 (-5.1, 10.8) |
| Age | 0.2 (0.1, 0.3) |
| Gender | 0.5 (-0.7, 1.8) |
| Disease duration | -0.1 (-0.4, 0.3) |
| JADAS at start of treatment | -0.9 (-0.9, -0.8) |

Number of patients in model: 711 (for whom both ethnicity and IMD were available). Coefficient is change in JADAS. A negative value indicates a greater improvement in JADAS compared to the reference group. IMD – Index of deprivation, comparing most deprived quintile to all others. Disease duration – time between diagnosis and commencement of methotrexate (years).
